# Editor's notes

**DOI:** 10.1093/nsr/nwz011

**Published:** 2019-01-24

**Authors:** Chung-I Wu

**Affiliations:** 1 School of Life Science, Sun Yat-Sen University, China; 2 Department of Ecology and Evolution, University of Chicago, USA; 3 Section Editor for Life Sciences at NSR

The two research articles from the Institute of Neuroscience of Chinese Academy of Sciences represent the new format for publishing significant findings as stated in an editorial of *National Science Review* [1].

The first article describes the generation of a group of gene-edited macaque monkeys by CRISPR/Cas9 editing of monkey embryos that deleted the expression of a core circadian regulatory transcription factor BMAL1. These monkeys exhibited the loss of sleep and psychosis-like phenotypes. The second article describes the cloning of a group of monkeys by somatic cell nuclear transfer (SCNT), using a juvenile gene-edited monkey from the first study that exhibits the most severe circadian-disorder phenotypes. These two pieces together demonstrate that a population of customized gene-edited macaque monkeys with uniform genetic background will soon be available for biomedical research.

The two articles went through the review and revision process thoroughly within 4 weeks. There is undoubtedly room for improving the two papers. For example, some reviewers inquire whether the five monkeys cloned by SCNT have been examined for the same array of phenotypes as the founder A6. To meet these requirements, the authors will have to delay the publications by several months. *NSR*'s philosophy is to avoid unnecessary lengthy delay in publishing significant findings, if the purpose of the delay is simply to wring out the last few percentage points of the results [2]. The timely publication of the main finding may in fact facilitate exhaustive investigations in follow-up work.
